# Hypertrophic cardiomyopathy with a large apical ventricular aneurysm and mural thrombus

**DOI:** 10.21542/gcsp.2018.9

**Published:** 2018-03-14

**Authors:** Munis Raza, Nagib Chalfoun, Abdallah Wissam, Hamza Hashmi, Richard McNamara

**Affiliations:** 1 Grand Rapids Medical Education Partners/Michigan State University Internal Medicine Residency, Grand Rapids, Michigan; 2 Frederik Meijer Heart and Vascular Institute, Spectrum Health, Grand Rapids, Michigan

## Abstract

Hypertrophic cardiomyopathy (HCM) is characterized by increased left ventricular wall thickness in the absence of any other identifiable cause of thickness. It predisposes the patient to increased risk of sudden cardiac death (SCD) due to fatal arrhythmias. Approximately 2% of the HCM patients have left ventricular apical aneurysm. CMR imaging is better in identifying this apical aneurysm as compared to echocardiogram. This apical aneurysm, which can be akinetic or dyskinetic, increases the risk of disease-related adverse events as compared to general HCM. These adverse disease-related events include SCD, thromboembolism, and symptoms of heart failure. We report a rare case of hypertrophic cardiomyopathy in association with Williams-Beuren Syndrome. On CMR imaging, patient was found to have a large apical aneurysm and mid-ventricular obstruction with underlying thrombus. He was started on oral anticoagulation, and ICD was recommended.

## Introduction

Hypertrophic cardiomyopathy (HCM) is defined as an unexplained increase in left ventricular (LV) wall thickness ≥ 15 mm associated with non-dilated ventricular chambers in the absence of another cardiac or systemic disease capable of producing the same magnitude of hypertrophy evident in a given patient^1^. Left ventricular apical aneurysm is being identified with increased frequency in HCM patients. This subset of HCM has a higher rate of adverse disease-related events compared to the general HCM patients. We present a case of hypertrophic cardiomyopathy with a large apical aneurysm and mural thrombus with mid-ventricular obstruction and cavity obliteration. This subset of hypertrophic cardiomyopathy has not been reported before in association with Williams-Beuren (WB) syndrome.

### The case

**Figure 1.**
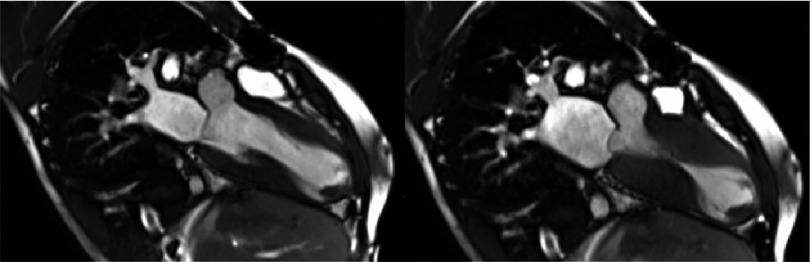
Two chamber view of the heart on cardiac MRI during diastole and systole. There is mid-cavity obliteration during systole; an apical aneurysm with an apical thrombus.

**Figure 2.**
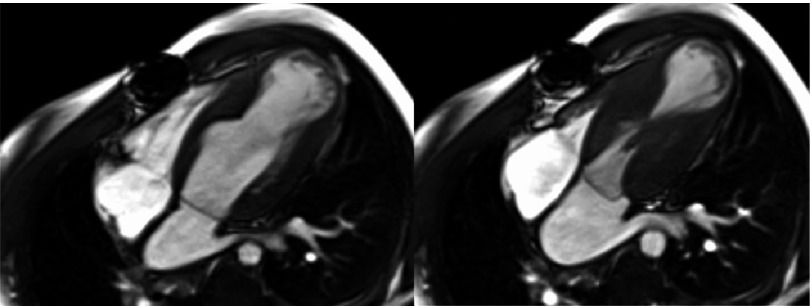
A four-chamber view showing mid-cavity obliteration during the systole. Showing the presence of apical aneurysm with an apical thrombus.

**Figure 3.**
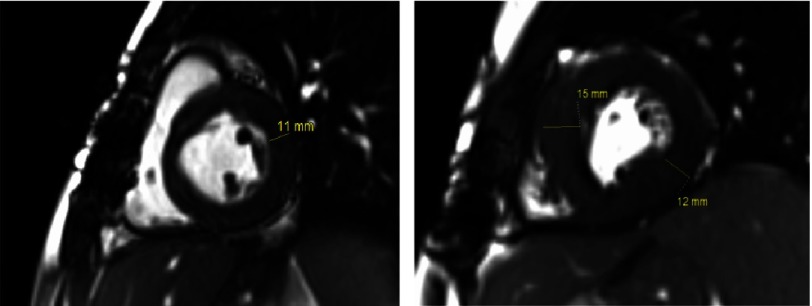
Showing normal thickness of base compared to the thickened mid-ventricular wall.

A 27-year-old man with known WB syndrome presented to the Emergency Department with fever three days prior to admission. Blood cultures were positive for Streptococcus mitis/Streptococcus oralis. To rule out infective endocarditis, a transthoracic echocardiogram (TTE) was obtained, which revealed a hyperdynamic left ventricle with mid-ventricular obstruction and cavity obliteration and distal akinesis. Cardiac magnetic resonance imaging (CMR) showed mid-segment left ventricular hypertrophy with wall thickness of 15 mm, a large apical aneurysm of 4 cm with 11 × 21 mm mural thrombus (Figures 1–3). Left ventricular systolic function was hyperdynamic; however, overall left ventricular ejection fraction was mildly reduced when the volume of the aneurysm was included in the volumetric calculations. There was patchy late gadolinium enhancement (LGE) of the hypertrophied segments and left ventricular aneurysm consistent with myocardial fibrosis.

The patient was discharged home to complete 14 days of IV antibiotics. He was started on oral anticoagulation with appropriate bridging for the left apical thrombus for an indefinite duration. An Implantable Cardiac Defibrillator (ICD) was recommended but the patient declined. Ambulatory ECG monitoring (Holter Monitor) showed one episode of non-sustained ventricular tachycardia and 19% premature ventricular complexes. He has no other major cardiovascular-related adverse events and has been maintained on oral anticoagulation.

## Discussion

We present a rare case of hypertrophic cardiomyopathy with a large apical aneurysm in association with WB Syndrome, which according to our knowledge has not been reported before. WBS is a multisystem genetic disorder, and sudden cardiac death is the most common cause of death in adult patients^2^. HCM is a genetically determined disease which is characterized by left ventricular hypertrophy of various morphologies, with a broad range of clinical manifestations and hemodynamic abnormalities. HCM is the most common cause of sudden cardiac death (SCD) in young adults. In general HCM carries an annual risk of SCD of approximately 1%. Chronic heart failure, arrhythmias and thromboembolism are common long-term manifestations with HCM^3^. The decision of ICD placement for primary prevention in these patients is based on the presence of one major risk factor, which include LV wall thickness ≥30 mm, non-sustained ventricular tachycardia (NSVT), family history of SCD, unexplained syncope and abnormal blood pressure response to exercise^4^.

With the advancement of cardiovascular imaging, LV apical aneurysm is being increasingly identified in HCM patients. LV apical aneurysm is defined as a discrete thin-walled most distal portion of LV chamber which can be akinetic or dyskinetic and connected to the cavity with a wide communication. Approximately 2% of HCM patients have been found to have an apical aneurysm. An apical aneurysm can be identified on echocardiogram with the use of contrast agent, but it is more reliably identified on CMR. There is LGE of the apical aneurysm on CMR due to the presence of myocardial scarring and fibrosis. The clinical significance of apical aneurysm has been debated but recent studies report an increased risk of mortality and morbidity as compared to general HCM patients. The adverse disease-related event rate for this subset of patients has been reported in one study to be 6.4%/year which is more than 3-fold increase as compared to general HCM patients^5^. These events include SCD, heart failure symptoms and thromboembolic events. The clinical significance of the size of aneurysm in relation to SCD remains unsettled.

Some studies^6^ have reported 5-fold increased risk of arrhythmia in these patients, which suggests LV aneurysm and associated fibrosis may itself represent one of the SCD risk factors. Myocardial scarring and fibrosis around the rim of an LV apical aneurysm represent the primary arrhythmogenic substrate^7^ for generation of ventricular arrhythmias (commonly monomorphic ventricular tachycardia) leading to SCD. Thromboembolic events have been reported to be 2-fold more frequent in these patients in comparison to general HCM patients. The apical aneurysm is thought to provide a structural nidus for intracavitary thrombus formation due to its akinetic/dyskinetic nature. Most of these events have been reported in patients with large or medium sized aneurysm in the presence of normal sinus rhythm, even with no evidence of intracavitary thrombus on CMR. Oral anticoagulation should be considered in these patients on a case-by-case basis.

## Conclusion

This case represents a unique presentation of hypertrophic cardiomyopathy with a large apical aneurysm and mural thrombus. This subset of patients with apical aneurysm appears to be at increased risk for sudden death, progressive heart failure symptoms and systemic thromboembolism. CMR is the optimal imaging technique to detect apical aneurysm and thrombus.
